# Cryoglobulinemic vasculitis triggered by *Staphylococcus aureus* endocarditis with chronic hepatitis C virus co-infection: a case report and literature review

**DOI:** 10.3389/fimmu.2024.1385086

**Published:** 2024-07-15

**Authors:** Céline Reinberg, Sébastien Vingerhoets, Olesya Pavlova, Emmanuella Guenova, Matthaios Papadimitriou-Olivgeris, Denis Comte

**Affiliations:** ^1^ Service of Internal Medicine, Lausanne University Hospital, University of Lausanne, Lausanne, Switzerland; ^2^ Service of Infectious Diseases, Lausanne University Hospital, University of Lausanne, Lausanne, Switzerland; ^3^ Service of Dermatology, Lausanne University Hospital, University of Lausanne, Lausanne, Switzerland

**Keywords:** case report, cryoglobulinemic vasculitis, endocarditis, *Staphylococcus aureus*, bacteremia, tricuspid valve

## Abstract

Infective endocarditis is a rare but life-threatening condition, occasionally linked to diverse immunologic manifestations, including mixed cryoglobulinemia. This can lead to cryoglobulinemic vasculitis, which has the potential for widespread organ damage. Although some cases have highlighted the relationship between infective endocarditis and cryoglobulinemic vasculitis, no comprehensive epidemiological evaluation or optimal treatment strategies have been advanced for such a combination. We present a case of methicillin-sensitive *Staphylococcus aureus* infective endocarditis associated with cryoglobulinemic vasculitis and conduct a literature review to compare management and outcomes in similar cases. Our patient presented with classical Meltzer’s triad and mild renal involvement. Cryoimmunofixation confirmed type III cryoglobulinemia, and serum cytokines showed elevated IL-6 levels. The differential diagnosis included infective endocarditis and chronic active hepatitis C virus infection. Rapid symptom resolution after antibiotic treatment identified infective endocarditis as the likely cause of cryoglobulinemic vasculitis. Our case and review of the literature highlight that early identification of the cause of cryoglobulinemic vasculitis is crucial for selecting appropriate treatment and preventing recurrence or morbidity.

## 1 Introduction

Infective endocarditis (IE) is a rare but life-threatening condition with high morbidity (1, 2). Over the years, IE has been associated with various immunologic manifestations, including cryoglobulinemia (3). Cryoglobulins are immunoglobulins that precipitate *in vitro* at temperature below 37°C and re-dissolve upon rewarming. Brouet classified cryoglobulins into three groups: Type I contains single monoclonal immunoglobulins (either IgM or IgG) and is often associated with B-cell lymphoproliferative disorders. Type II commonly combines monoclonal IgM with rheumatoid factor activity binding to the Fc portion of polyclonal IgG. Type III consists of a mixture of polyclonal immunoglobulins (4). Both type II and III, often collectively referred to as mixed cryoglobulinemia, constitute 80–85% of cases and are usually the result of infectious disease, malignancy and auto-immune disease (mainly Sjögren’s syndrome and systemic lupus erythematosus). Cryoglobulinemia is mostly asymptomatic. However, mixed cryoglobulinemia can lead to a systemic immune-complex-mediated vasculitis named cryoglobulinemic vasculitis (CV), which has the potential for widespread organ damage (5). Detection of cryoglobulinemia is biologically confirmed by the precipitation of serum cryoglobulins at 4°C for up to 7 days with levels > 0.05g/L in two separate determinations. The composition of the cryoprecipitate is typically analyzed using immunoelectrophoresis and immunofixation techniques. Additionally, the cryocrit level is assessed (6, 7), although its direct correlation with clinical manifestations of the disease remains debated (8, 9). Due to the complex diagnostic process to isolate cryoglobulins and variable clinical manifestations, the exact prevalence of cryoglobulinemia remains elusive but is estimated to affect around 1 in 100’000 individuals (4, 7, 10). Recently, classification criteria for HCV-related and HCV-unrelated CV have been established and validated (11).

Hepatitis C virus (HCV) infection is associated with most cases of mixed cryoglobulinemia (4). Although some cases have highlighted the relationship between IE and CV, no comprehensive epidemiological evaluation has yet been undertaken. Similarly, no universally accepted approach to managing this combination exists, and no randomized trials have explored optimal treatment strategies.

In this report, we present a relevant case and review the literature (**Supplementary Table 1**) to compare management and outcomes in similar cases. Case report information has been organized as a chronological timeline (**Supplementary Figure 1**).

## 2 Case report

**Figure 1:**
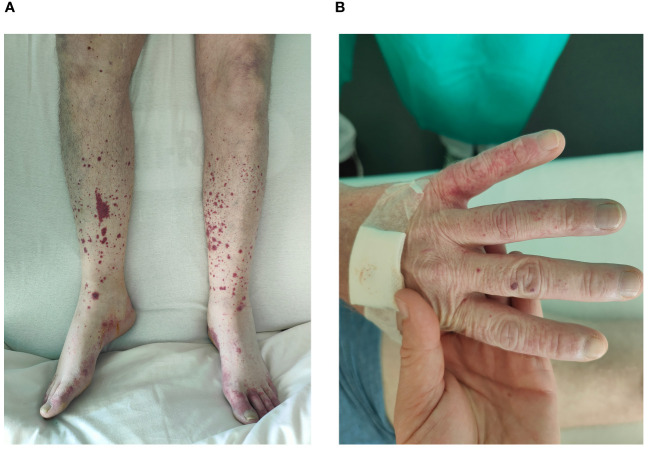
Clinical manifestations of cryoglobulinemic vasculitis in our patient. **(A)** Lower limbs palpable purpura. **(B)** Raynaud’s phenomenon and purpuric rash.

A 57-year-old patient with a history of intravenous drug usage and chronic HCV infection presented asthenia, myalgia, back pain, and elbow arthralgia accompanied by a palpable purpura on the lower limbs and hands (**Figure 1A**) and a new Raynaud’s phenomenon (**Figure 1B**). Symptoms began two weeks earlier, following an episode of acute gastroenteritis. In the past five days, the patient experienced progressive dyspnea with dry cough, muscle weakness and nocturnal burning sensations in the lower limbs.

Upon admission, the patient had a 38.1°C fever and a subacute altered mental state, but no other neurologic deficits. Pallesthesia tests were normal. The cardiologic examination revealed lower limb oedema and a new 2/6 systolic murmur at the lower left sternal edge. Laboratory studies showed increased inflammatory parameters: WBC at 12.8 x10^9^/L, predominantly segmented neutrophils, CRP at 223 mg/L, and erythrocyte sedimentation rate of 57 mm/h. The complete blood cell count revealed normochromic microcytic hyporegenerative anemia (hemoglobin 101 g/L) and mild thrombocytopenia (104 x10^9^/L), with no sign of thrombotic microangiopathy. Coagulation tests indicated disseminated intravascular coagulation (PT 47%, aPTT 48 s, INR 1.5). The Direct Coombs test was positive. Creatinine was 1.3 mg/dL (119 μmol/l; CKPD-EPI glomerular filtration rate of 58 mL/min/1.73). Liver function tests were normal. Urinalysis showed proteinuria (360 mg/24h), microhematuria, and leukocyturia without acanthocytes.

**Figure 2:**
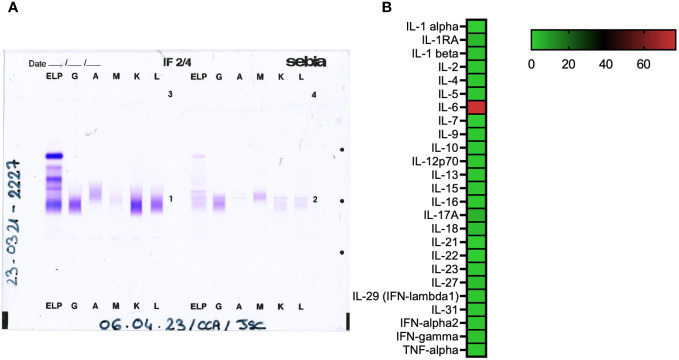
**(A)** Cryoimmunofixation: on the left, serum migration and on the right, cryoprecipitate type III. **(B)** Relative serum cytokine levels analyzed using the Luminex Technique.

Blood cultures were positive for methicillin-sensitive *Staphylococcus aureus*. Serologic tests indicated a resolved hepatitis B infection and a chronic hepatitis C infection (genotype 4d) with a positive HCV viremia. Abdominal ultrasound and computerized tomography (CT) showed no signs of hepatic fibrosis, no portal hypertension or splenomegaly. Cryoimmunofixation (**Figure 2A**) was consistent with type III cryoglobulinemia (0.238 g/l, polyclonal IgG). Immunological analysis showed a low serum C4 level (0.12 g/L), but normal C3c level and rheumatoid factor. Serum cytokines analysis by Luminex revealed a marked elevation of IL-6 (540 pg/ml) without significant changes in other cytokines (**Figure 2B**).

**Figure 3:**
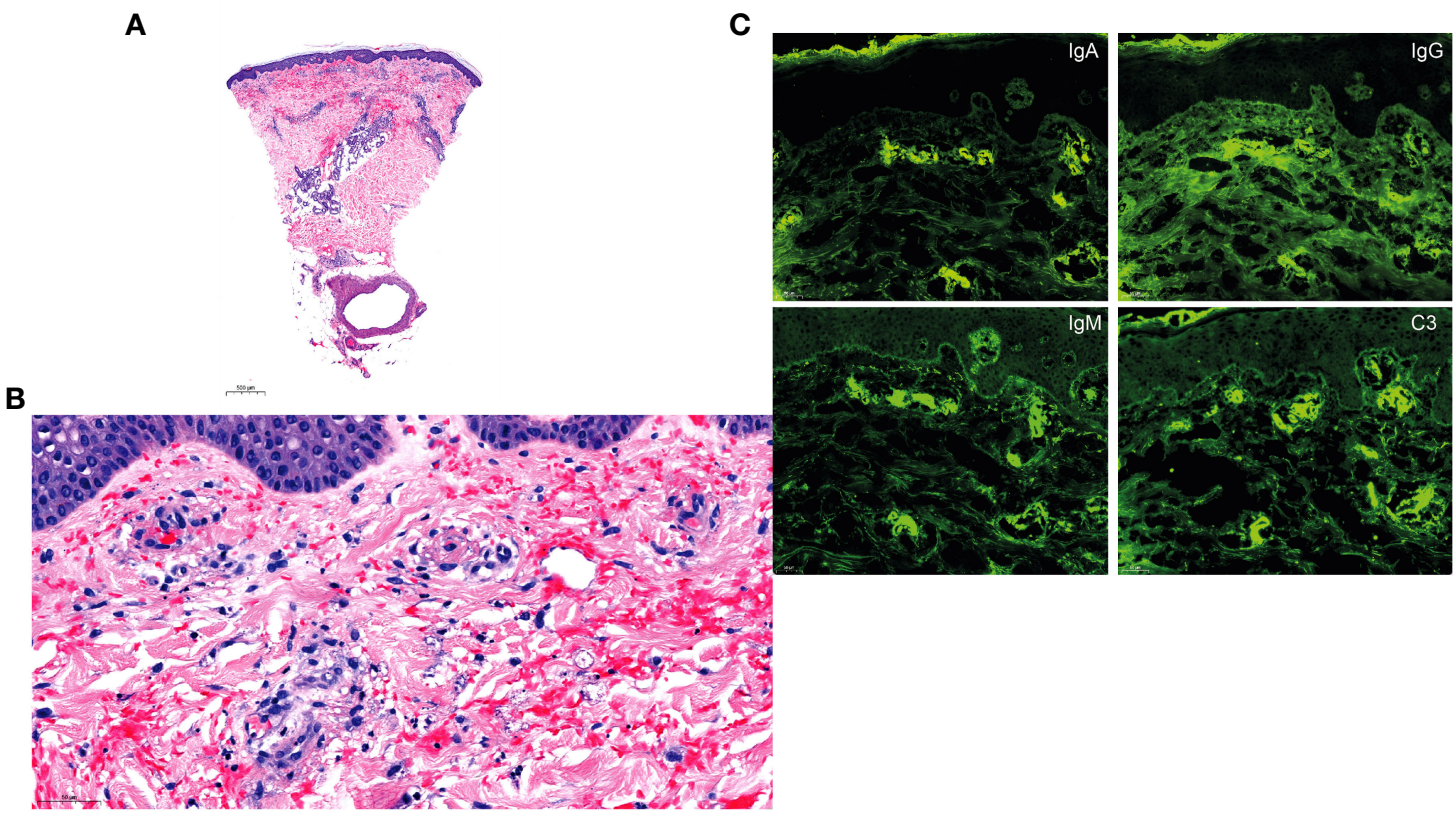
Histology and direct immunofluorescence. **(A, B)** H&E staining of a skin biopsy of the purpuric lesion on the left lower limb revealing mixed perivascular and interstitial inflammatory infiltrate around the capillaries of the superficial and deep dermis, composed of altered neutrophils with some eosinophils. Note the leukocytoclasia with nuclear dust, frequent capillary thromboses, parietal alterations with endothelial clarification and vacuolisation, fibrinoid necrosis with intraluminal fibrin deposition, and vessel wall disruption with red cell extravasation in the superficial, middle and deep dermis. **(C)** Direct immunofluorescence with deposits of IgM, IgG, IgA and C3 along the dermoepidermal junction and the superficial vasculature.

A punch biopsy of the purpuric lesion on the left lower limb revealed leukocytoclastic vasculitis with perivascular and interstitial inflammatory infiltrate around the capillaries of the superficial and deep dermis, mostly composed of altered neutrophils, with some eosinophils. We observed leukocytoclasia with nuclear dust, frequent capillary thromboses, parietal alterations with endothelial clarification and vacuolisation, fibrinoid necrosis, and vessel wall disruption with red cell extravasation (**Figures 3A, B**). No image of septic emboli was identified. Direct immunofluorescence showed IgM, IgG, IgA and C3 deposits along the dermoepidermal junction and in the vessel walls of the superficial skin vasculature (**Figure 3C**). Skin culture was negative. Fundoscopy showed no pathologic lesions. IE of the tricuspid valve was confirmed by transthoracic echocardiography, revealing a 29mm vegetation associated with mild tricuspid insufficiency. Chest CT detected multiple pulmonary abscesses from septic pulmonary emboli and an acute left upper lobar pulmonary embolism. MRIs could not be completed due to claustrophobia, but showed no cerebral vasculitis or septic emboli, and confirmed, however, a T2 hypersignal at the cervical vertebra C6-C7, suggestive of spondylodiscitis.

The patient received initial empirical treatment of intravenous ceftriaxone and clarithromycin, later adjusted to flucloxacillin monotherapy based on blood culture results. The patient underwent tricuspid valve replacement. The pulmonary embolism was treated with unfractionated heparin. A 6-week course of intravenous flucloxacillin was administered for suspected C6-C7 spondylodiscitis. Remarkably, skin lesions, muscle weakness, nocturnal burning sensations and kidney symptoms completely resolved within days of starting antibiotic therapy. Eighteen months later, without hepatitis C treatment, there was no recurrence of skin lesions.

## 3 Methodology

We conducted a systemic literature search via PubMed^®^ using the keywords “Endocarditis” AND “Cryoglobulin” or “Cryoglobulinemia” with or without “Vasculitis” and “Glomerulonephritis”.

The inclusion criteria for articles were as follows:

- Case report or case series with available full-text article:
- Definite IE as defined by the modified Duke criteria in the 2023 European Society of Cardiology guidelines (12)

And

- Presence of documented cryoglobulinemia in biological work-up

And

- Evidence of compatible end-organ damage such as vasculitis or acute kidney injury.

And

- No prior history of immunologic or rheumatologic disease.
- No alternative diagnosis explaining end organ damage.

Our systematic research identified 21 articles, with 19 meeting the inclusion criteria (13–30).

## 4 Discussion

This clinical case report highlights classical symptoms of cryoglobulinemic vasculitis, including purpura, weakness and arthralgia, collectively referred to as Meltzer’s triad. Additionally, the patient presented with Raynaud’s phenomenon, occurring in about 25% of affected patients (5). Neurologic symptoms were limited to superficial sensation abnormalities without signs of polyneuropathy. Renal manifestations were mild, with acute kidney injury, proteinuria and micro-hematuria. Interestingly, the proteinuria was non-selective, with albuminuria accounting for only 2.7%, implying no glomerular involvement. Given the rapid recovery of renal function, no renal biopsy was necessary. As previously described (28), acute kidney injury is a prominent organ manifestation of cryoglobulinemia. Importantly, our patient did not encounter life-threatening complications commonly associated with CV, such as glomerulonephritis, alveolar hemorrhage or intestinal ischemia (4, 5). The dry cough and dyspnea were primarily attributed to septic pulmonary emboli, secondary to IE, and pulmonary embolism.

Type III cryoglobulinemia accounts for 25–30% of cases (10). Non-HCV infectious mixed CV more frequently affects men in their sixth decade, aligning with our patient’s profile (28).

Epidemiologically, comparing cases from this review (**Supplementary Table 1**) with data from the institutional IE cohort reveals a higher proportion of intracellular pathogens (40% *versus* 1–2% in the cohort) and a lower proportion of *S. aureus* (20% *versus* 39%) (31). IE caused by intracellular pathogens typically follows a subacute or chronic course, leading to diagnostic delays due to sterile blood cultures and nonspecific symptoms. In contrast, *S. aureus* usually triggers acute IE with frequent classical symptoms and positive blood cultures, leading to a quicker diagnostic.

The delay in diagnosing subacute or chronic IE increases the risk of developing immunological manifestations, suggesting that a requisite time interval is needed for generating a polyclonal humoral response and developing mixed cryoglobulinemia. Interestingly, the higher frequency of immunological manifestations in subacute IE was previously reported (28). In contrast to left-sided IE, right-sided IE, as in our case, has a milder presentation, allowing for the development of the observed immunological response.

Our patient’s differential diagnosis for CV included IE and chronic active HCV infection. Rapid symptom resolution post-antibiotic treatment identified IE as the probable disease catalyst. Eighteen months later, despite ongoing HCV viremia, there was no recurrence of skin lesions. The co-infection prevalence between chronic HCV infection and IE among HCV patients was lower than expected, possibly due to reporting bias. Since HCV infection is the primary cause of mixed cryoglobulinemia with a well-established therapeutic management, some authors have excluded such cases to focus on other potential underlying etiologies with different onset of presentation, management and prognosis (7, 28).

Identifying CV underlying etiology is essential to select the appropriate treatment and avoid an increase in recurrence, morbidity or mortality. Misdiagnosed IE-related cryoglobulinemia treated with single immunosuppressive therapy led to relapsing (21, 27, 30). For many cases in our review (**Supplementary Table 1**), including our patient, when bacterial infection was the underlying cause, anti-infective treatment alone was sufficient to result in complete clinical remission (32).

In cases of mixed cryoglobulinemia not caused by infection but rather associated with auto-immune diseases, immunosuppressive therapy has been found to be an efficient treatment (5, 7). Using biological B cell depleting agents, such as rituximab, eventually combined with glucocorticoids, has shown significant efficacy in patients with severe manifestations or refractory forms of CV, as a second-line therapy, where antibiotics alone fail to control symptoms (19, 28). Plasma exchange can also be used in life-threatening conditions (4, 6, 28). We observed an isolated elevation of IL-6, an early marker of inflammation (33), without significant changes in other cytokines usually seen in context of sepsis or bacterial infections (34, 35). Elevated IL-6 in CV related to HCV (36) and severe *S. aureus* infections (37) suggests potential for IL-6 targeted therapy, although its use in rituximab-refractory CV cases has been anecdotal (38).

While our patient had no residual cryoglobulinemia symptoms 18 months after IE resolution, cryoglobulins were still detectable at the 6-month follow-up, likely due to chronic active hepatitis C. Unfortunately, treatment was unfeasible due to psychiatric morbidity and lack of adherence, making outpatient care unfeasible even for directly observed therapy.

The prevalence of circulating cryoglobulins in the HCV-infected population is 40–60% but only 5–15% will develop CV (8, 9). In this review (**Supplementary Table 1**), most patients had negative cryoglobulinemia following IE treatment. Persistent cryoglobulinemia requires considering ongoing co-infection or immunological disorder.

This review had limitations due to the rarity of these diseases, heterogeneous CV presentations, and complex detection and diagnosis processes, leading to various inclusion criteria and unidentified immunochemical types. Some studies focusing on non-HCV infectious CV might have missed some IE and CV associations due to reporting bias.

## 5 Conclusion

Our case report aligns with previous studies considering clinical presentation, evolution, epidemiological and demographic findings of CV associated with IE. Humoral response in mixed cryoglobulinemia requires sufficient time to develop, as suggested by the higher prevalence of CV in subacute or chronic infections, such as subacute IE due to intracellular pathogens. Treating the underlying etiology frequently leads to complete remission, highlighting the importance of identifying the cause of cryoglobulinemia (39, 40). Persistence of cryoglobulinemia post-IE cure, even if asymptomatic, should raise the question of another associated condition.

## 6 Recommendations

- Always investigate the underlying cause of cryoglobulinemia and treat it if identified.
- In the presence of mixed type II or III (polyclonal) cryoglobulinemia, consider potential subacute or chronic infections, particularly subacute IE.
- For suspected IE in cases of mixed type II or III cryoglobulinemia, be aware of the higher prevalence of atypical, intracellular pathogens.
- Treating the underlying infection in CV due to IE typically leads to complete clinical remission.
- When a patient with IE presents with skin manifestations, such as petechiae, palpable purpura, livedo reticularis, together with a kidney injury, suspect vasculitis, and, particularly, cryoglobulinemia.-
- Persistence of cryoglobulinemia post-IE antibiotic cure, even if asymptomatic, should raise the question of another associated condition, including infection, immunological disorder or hematological disease.

## Data availability statement

The raw data supporting the conclusions of this article will be made available by the authors, without undue reservation.

## Ethics statement

The studies involving humans were approved by the ethics committee of the Canton of Vaud (CER-VD 2017 – 02137). The studies were conducted in accordance with the local legislation and institutional requirements. Written informed consent for participation was not required from the participants or the participants’ legal guardians/next of kin in accordance with the national legislation and institutional requirements. Written informed consent was obtained from the individual(s) for the publication of any potentially identifiable images or data included in this article.

## Author contributions

CR: Writing – original draft, Writing – review & editing, Conceptualization, Methodology, Validation, Visualization. SV: Writing – original draft, Writing – review & editing, Methodology, Validation. OP: Writing – review & editing. EG: Writing – review & editing. MP-O: Writing – review & editing. DC: Supervision, Writing – original draft, Writing – review & editing, Conceptualization, Methodology, Validation, Visualization.
