# Supplementary material for: Cryoglobulinemic vasculitis triggered by Staphylococcus aureus endocarditis with chronic hepatitis C virus co-infection: a case report and literature review
Source: Front Immunol. 2024 Jul 15;15:1385086. doi: 10.3389/fimmu.2024.1385086 (PMC11284083; doi:10.3389/fimmu.2024.1385086)
Supplement: Supplementary file 1 [file DataSheet_1.docx]

Supplementary Material

**Cryoglobulinemic vasculitis triggered by *Staphylococcus aureus* endocarditis with chronic hepatitis C virus co-infection: a case report and literature review**

Céline Reinberg^1^*, Sébastien Vingerhoets^2^, Olesya Pavlova^3^, Emmanuella Guenova^3,^ Matthaios Papadimitriou-Olivgeris^2^, Denis Comte^1^

^1^ Service of Internal Medicine, Lausanne University Hospital, University of Lausanne, Lausanne, Switzerland

^2^ Service of Infectious Diseases, Lausanne University Hospital, University of Lausanne, Lausanne, Switzerland

^3^ Service of Dermatology, Lausanne University Hospital, University of Lausanne, Lausanne, Switzerland

*** Correspondence:** Corresponding Author: celine.reinberg@chuv.ch

# Supplementary Table

**Supplementary Table 1.** Summary of the reported cases of mixed cryoglobulinemia due to bacterial infection

| **Ref.** | | **Documented bacterial etiology** | | **Valve** | **Hepatitis or HIV**  **(N/ B/ C/ HIV)** | | **Laboratory studies** | | **Organ damage** | | **Use of IST** | | **Outcome of the CV** | |
| --- | --- | --- | --- | --- | --- | --- | --- | --- | --- | --- | --- | --- | --- | --- |
| (13) Collazos J. and al | *A.actinomycetem comitans* | | n.P | | | N | | Cryo Type III,  Low C3,C4 | | AKIN, peripheral vasculitis | | none | | cured |
| (14) Nallarajah J. and al. | *B. cereus* | | n.M | | | N | | Cryo n/s,  Low C4 | | AKIN | | none | | cured |
| (15) Georgievskaya Z. and al. | *B. henselae* | | p.P | | | N | | Cryo Type ll,  Low C4 | | AKIN | | none | | improvement |
| (16) Babiker A. and al. | *B. henselae* | | p.A | | | N | | Cryo Type lll,  Low C3, C4 | | AKIN (hemodialysis) | | glucocorticoids, plasmapheresis | | improvement |
| (17)  Vivekanantham A. and al. | *B.henselae* | | p.P | | | N | | Cryo Type lll | | AKIN, peripheral vasculitis | | prednisolone | | cured |
| (18) Rafailidis P. and al. | *C. burnetii* | | p.M | | | N | | Cryo Type ll,  Low C4 | | peripheral vasculitis | | prednisolone | | cured |
| (19) Hawkins K. and al. | *C. burnetii* | | n.M | | | N | | Cryo Type ll,  Low C4 | | AKIN, PPN, peripheral vasculitis | | glucocorticoids Rituximab | | cured after adjuvant anti-CD20 |
| (20) Vacher-Coponat H. and al. | *C. burnetii* | | p.M | | | n/a | | Cryo Type ll | | AKIN | | none | | cured |
| (21) Sim B. and al. | *G. adiacens* | | n.A | | | N | | Cryo Type ll | | peripheral vasculitis | | glucocorticoids, methotrexate | | relapse after IST alone then cured |
| (22) Liu K. and al. | *S. aureus* | | n.A | | | N | | Cryo n/s | | AKIN, peripheral vasculitis | | glucocorticoids | | cured |
| (23) Josephson and al. | *S. aureus* | | n.T | | | N | | Cryo Type lll,  Low C4 | | peripheral vasculitis | | glucocorticoids | | improvement |
| (23) Josephson and al. | *S. aureus* | | n.T | | | C | | Cryo Type ll,  Low C3 | | AKIN, peripheral vasculitis | | none | | cured |
| (24) Bele D. and al. | *S. cristatus* | | n.M | | | N | | Mixed Cryo,  Low C3 | | AKIN | | IVIg, glucocorticoids | | cured |
| (25) Orfila C. and al. | *S. mitis* | | n.M | | | n/a | | Cryo n/s | | AKIN, peripheral vasculitis | | none | | improvement |
| (26) Zito A. and al. | *S. viridans* | | n.A | | | C | | Mixed Cryo,  Low C4 | | peripheral vasculitis | | none | | cured |
| (27) Agarwal A. and al. | *Streptococcus spp* | | n.A/n.P/VSD | | | N | | Cryo Type lll,  Low C3, C4 | | AKIN, peripheral vasculitis | | glucocorticoids, CP | | relapse after IST alone then improvement |
| (28) Terrier B. and al. | *Streptococcus n/s* | | n/s | | | N | | Cryo Type III,  Normal C3,C4 | | AKIN, peripheral vasculitis | | none | | cured |
| (29) Kodo K. and al. | *Streptococcus pyogenes* | | none | | | N | | Cryo Type III,  Low C3 | | AKIN (hemodialysis), peripheral vasculitis | | glucocorticoids,  azathioprine | | fatal relapse after IST alone |
| (30) Messiaen T. and al. | sterile | | n.A | | | N | | Cryo Type III,  Low C3 | | AKIN, peripheral vasculitis | | glucocorticoids, CP | | relapse after IST alone then improvement |
| Our case report | *S. aureus* | | n.T | | | C | | Cryo Type III,  Normal C3, Low C4 | | AKIN, peripheral vasculitis | | none | | cured |

# n.M, native mitral valve; n.P, native pulmonary valve; n.A, native aortic valve; n.T, native tricuspid valve; p.M, prothetic mitral valve; p.P, prothetic pulmonary valve ; p.A prothetic aortic valve; VSD, ventricular septal defect; n/s, non specified; N, no; n/a, non assessed; B, positive hepatitis B; C, positive hepatitis C; HIV, human immunodeficiency virus; Cryo, Cryoglobulinemia; CV, cryoglobulinemic vasculitis; AKIN, acute renal injury; CP, cyclophosphamide; IVIg, intravenous immunoglobulin therapy; IST, immunosuppressive therapy

# Supplementary Figure

**Supplementary Figure 1.** Chronological timeline of the case report

**
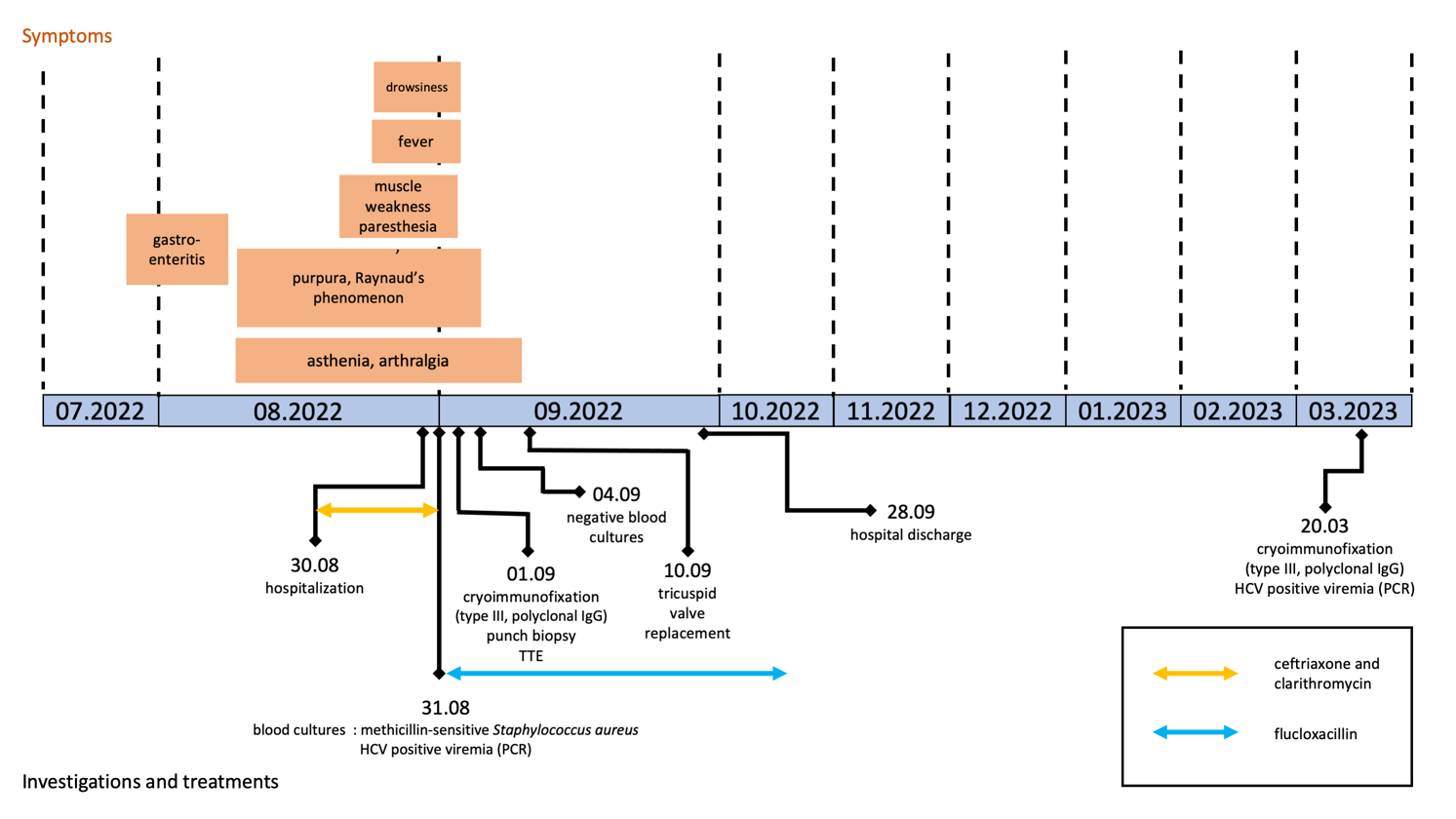
**
